# Role of Matrix Metalloproteinases 7 in the Pathogenesis of Laryngopharyngeal Reflux: Decreased E-cadherin in Acid exposed Primary Human Pharyngeal Epithelial Cells

**DOI:** 10.3390/ijms20215276

**Published:** 2019-10-24

**Authors:** Nu-Ri Im, Doh Young Lee, Byoungjae Kim, Jian Kim, Kwang-Yoon Jung, Tae Hoon Kim, Seung-Kuk Baek

**Affiliations:** 1 Department of Otorhinolaryngology-Head and Neck Surgery, College of Medicine, Korea University, Seoul 02841, Korea; snwksnwksnwk@hanmail.net (N.-R.I.); autru222@gmail.com (B.K.); 1023kdh@naver.com (J.K.); kyjungmd@gmail.com (K.-Y.J.); 2 Department of Otorhinolaryngology-Head and Neck Surgery, Seoul National University Boramae Medical Center, Seoul 07061, Korea; gedo0212@naver.com; 3 Neuroscience research institute, College of Medicine, Korea University, Seoul 02841, Korea

**Keywords:** E-cadherin, matrix metalloproteinase, laryngopharyngeal reflux, acid, permeability

## Abstract

Cleavage of E-cadherin and the resultant weakness in the cell-cell links in the laryngeal epithelium lining is induced by exposure to acidic contents of the refluxate. Herein, we aimed to evaluate the role of matrix metalloproteinases (MMPs) in inducing E-cadherin level changes following acid exposure to the human pharyngeal mucosal cells. E-cadherin levels were inversely correlated with the duration of acid exposure. Treatment with actinonin, a broad MMP inhibitor, inhibited this change. Immunocytochemical staining and transepithelial permeability test revealed that the cell surface staining of E-cadherin decreased and transepithelial permeability increased after acid exposure, which was significantly inhibited by the MMP inhibitor. Among the various MMPs analyzed, the mRNA for MMP-7 in the cellular component was upregulated, and the secretion and enzymatic activity of MMP-7 in the culture media increased with the acid treatment. Consequently, MMP-7 plays a significant role in the degradation of E-cadherin after exposure to a relatively weak acidic condition that would be similar to the physiologic condition that occurs in Laryngopharyngeal reflux disease patients.

## 1. Introduction

Laryngopharyngeal reflux disease (LPRD) is an extraesophageal variant of gastroesophageal reflux disease (GERD) characterized by dysphonia, globus pharyngeus, hoarseness, recurrent throat clearing, and chronic cough. LPR is estimated to account for 10% of all ear, nose, and throat (ENT) clinic patients and 50% of patients with voice complaints [1]. The pathophysiology and objective diagnosis of LPRD are both still unclear, even though the incidence of LPRD has increased dramatically over recent years [2]. The most commonly accepted theory regarding the onset of LPRD is that its symptoms are a result of direct alteration of the laryngeal mucosa by gastric acid reflux, and due to this cellular origin of disease, LPRD is considered a different disease from the classic gastroesophageal reflux disease [3]. A diagnosis of LPRD can be established through a questionnaire for specific symptoms, a videolaryngoscopic evaluation of the larynx, or double-probe pH monitoring [4,5,6]. Ambulatory, 24-h double-probe (pharyngeal and esophageal) pH monitoring is both highly sensitive and specific for the diagnosis of LPRD [7,8]. However, it is not widely available in clinical practice due to its inconvenience and relatively high cost compared to more easily accessible videolaryngoscopic examination [9].

Although standard therapy consists of high-dose proton pump inhibitor (PPI) therapy [10], there is insufficient evidence to conclude that treatment with PPIs is superior to a placebo in randomized trials [11]. In addition, achieving a clinical response to such treatment with improvement of symptoms is relatively poor [12]. As such, the currently suggested pathophysiology does not adequately explain the pathogenesis of LPRD or the inconsistencies in the treatment response. However, given that the luminal environment of the pharynx is pH-neutral at 7.0 and the stomach secretes acid at a pH of 1.5 to 2.0, acid reflux into the laryngopharyngeal epithelium can lead to a significant decrease in pH. Epithelial damage can occur from this drop in pH or to exposure to noxious elements in the refluxate including pepsin, bile salts, and pancreatic enzymes [13]. To date, studies on LPRD have shown that acid reflux causes impairment of the larynx/hypopharynx, but the effects of acid on the larynx/hypopharynx are still unclear [14]. Even in the recent reflux study of the epithelial cell in vitro model, there is only a report on the formation, but the determination of the mechanism is still lacking [15]. E-cadherin is a component of the adherens junction. The adherens junctions along with tight junctions and desmosomes are important in junctional barrier formation in most epithelia, including that of the laryngopharynx [16]. It is a crucial epithelial structure that forms the permeability barrier that blocks the passive diffusion of most solutes between the luminal and interstitial spaces [17]. It is able to bind to the cytoskeleton through catenins necessary for the full adhesive function of cells. E-cadherin has an integral role in establishing junctional resistance and controlling junctional permeability [18]. Previous studies on the relationship between E-cadherin and LPR have shown that E-cadherin expression decreased in LPR patients [19]. An increase in junctional permeability in the laryngopharynx in LPRD can be due to disruption of protein bridge formation, and such an increase can also be due to various enzymes including matrix metalloproteinase (MMP), known to breakdown and fragment E-cadherin. Similarly, we have reported a preliminary study that E-cadherin in human nasal mucosa can be disrupted by acid exposure and that MMP-inhibitors can prevent such destruction [20]. In this study, we aimed to evaluate changes in E-cadherin in the pharyngeal mucosa after acid exposure and to assess the pathophysiologic mechanism associated with E-cadherin cleavage and MMP through in vitro models of human-derived pharyngeal mucosa.

## 2. Results

### 2.1. E-cadherin Cleavage by Acid Exposure in Pharyngeal Mucosal Epithelial Cells is Inhibited by MMP Inhibitor

**Figure 1:**
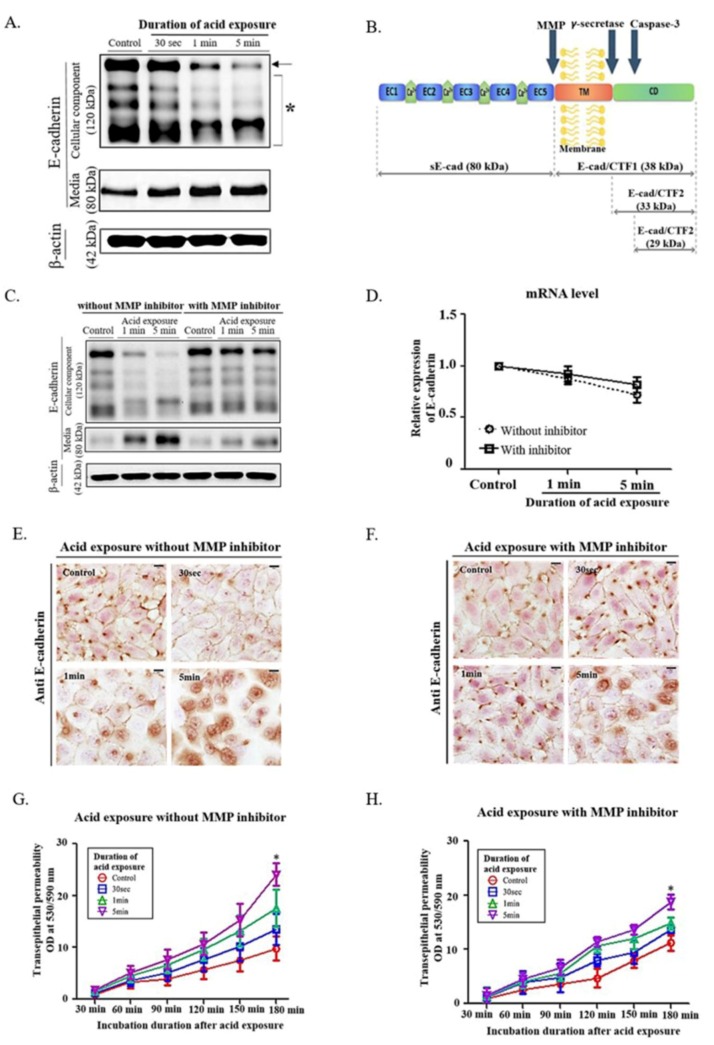
Effect of MMP inhibitor on changes of E-cadherin cleavage in human pharyngeal mucosal epithelial cells exposed to acidic media. (**A**) Evaluation of culture media shows an increase in levels of soluble 80-kDa E-cadherin (sE-cad) that is negatively correlated with the expression of full-length E-cadherin in the cell portions (arrow). Evidence of several cleavages is observed in the cellular component, and an increase in sE-cad is the most prominent change (asterisk). (**B**) Cleavage of full-length E-cadherin by MMPs generates sE-cad. CTF, C-terminal fragment; CD, cytoplasmic domain; EC, extracellular cadherin domain; TM, transmembrane domain; yellow structure, cell membrane. (**C**) Low expression of E-cadherin and high expression of sE-cad after acid exposure are significantly reversed by MMP inhibitor treatment. (**D**) mRNA level of E-cadherin is not changed irrespective of acid exposure and MMP inhibitor treatment. (**E**,**F**) Following acid exposure, the E-cadherin expression in the cell-cell junction is reduced, which is inhibited by MMP inhibitors. (bar, 50 µm) (**G**,**H**) The slope of the transepithelial permeability increases as acid exposure time increases but is inhibited by MMP inhibitor treatment. The permeability decreases significantly by MMP inhibitors at 180 min of incubation (* *p* < 0.05).

Based on our previous studies [20], we exposed human pharyngeal mucosal epithelial cells (Supplementary Figure S1) to pH-4 medium for 30 s, 1, 5 min, followed by washout and replacement of the medium with regular medium, and harvested the cells and the culture media after 24 h of non-acidic incubation in regular medium (Supplementary Figure S2A–C). As acid exposure time increased, the cleavage of full-length E-cadherin in the cell layer was enhanced, which was confirmed by increase of sE-cad (soluble 80-kDa E-cadherin) in the culture media (Figure 1A, Supplementary Figure S2D).

As cleavage of full-length E-cadherin into sE-cad is known to be enhanced by MMPs (Figure 1B), [21] the treatment of MMP inhibitor significantly reduced the cleavage of E-cadherin by acid exposure (Figure 1C, Supplementary Figure S2E). However, the mRNA level of E-cadherin was not changed by MMP inhibitors (Figure 1D), indicating that the reduced full-length E-cadherin was caused solely by cleavage.

Like our previous nasal epithelial study [20], immunocytochemical staining and transepithelial permeability test in pharyngeal cells showed that the increased permeability with the reduced cell-cell interaction was recovered by the treatment of MMP inhibitor (Figure 1E–H), suggesting that the MMP inhibitor decreases acid-induced permeability and maintains cell-cell interactions by blocking further cleavage of E-cadherin by MMPs.

### 2.2. Inhibition of MMP-7 Decreased the Acid-Related E-cadherin Cleavage of Pharyngeal Epithelial Cells

**Figure 2:**
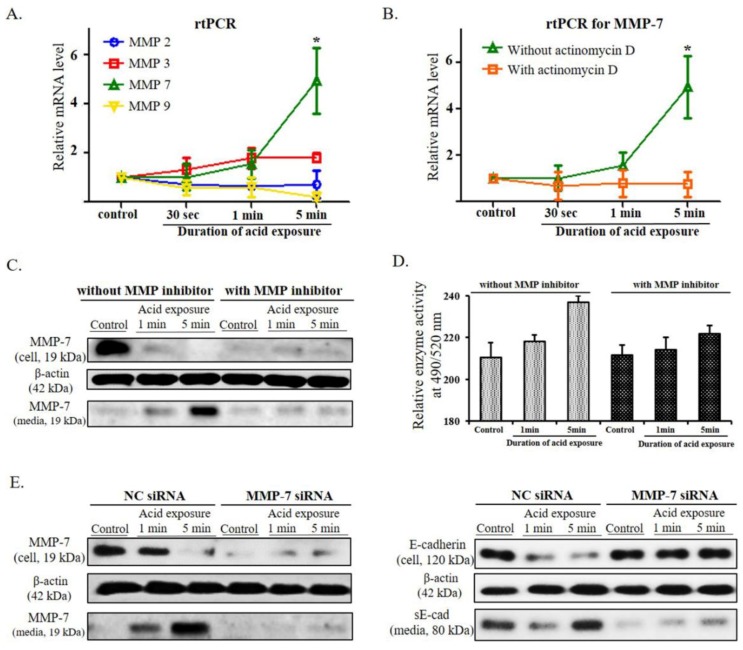
Changes in the expression of MMPs after acid exposure and changes in the expression and enzyme activity of MMP-7 according to MMP inhibitor treatment and MMP-7 knockdown. (**A**) As the duration of exposure to acid increases, the mRNA expression of MMP-2 and MMP-9 decrease, and that of MMP-3 and MMP-7 increase. The decrease in mRNA expression for MMP-9 and the increase in mRNA expression for MMP-7 at 5 min of acid exposure were particularly significant (* *p* < 0.05), while the changes in MMP-3 show an increasing tendency without a statistical significance. (**B**) When treated with actinomycin D, the mRNA expression of MMP-7 is not increased. (**C**) The intracellular decrease (cell) and extracellular increase (media) of the active forms of MMP-7 are elicited by increases in acid exposure time, while these changes are not identified after treatment with an MMP inhibitor. (**D**) As the cells are exposed to acid for longer periods of time, the enzyme activity of MMP-7 on the 5-FAM/QXL^TM^ 520 FRET peptide significantly decreases after treatment with an MMP inhibitor. (**E**) The expression of the active form of MMP-7 markedly decreases via the MMP-7 siRNA knockdown compared with that of negative control (NC siRNA). MMP-7 siRNA- transfected cells do not exhibit a decrease in the level of cellular E-cadherin or an increase in the level of sE-cad in culture media after acid exposure.

To investigate the effect of MMPs on E-cadherin cleavage in acidic environments, reverse transcription polymerase chain reaction (RT-PCR) was performed on MMP-2, 3, 7, and 9 that are known to be associated with E-cadherin cleavage [22]. Whereas mRNA expression of MMP-2, 3, and 9 not changed, MMP-7 significantly increased after acid treatment of the cells (*p* < 0.05) (Figure 2A). When actinomycin D, an intercalating transcription inhibitor, was used to treat the cultured cells, the level of the MMP-7 mRNA was significantly decreased (*p* < 0.05), indicating that the transcription of MMP-7 occurred during the 24 h of incubation after acid exposure. (Figure 2B).

At the protein level, the intracellular levels of the active form of MMP-7 were reduced as the duration of the exposure to acid increased, and correspondingly, the levels of that in the culture media increased. This change in MMP-7 levels was not elicited when the MMP inhibitor was applied to the acid-treated cell culture (Figure 2C, Supplementary Figure S3A). To evaluate the protein function itself, the enzymatic activities of MMP-7 was analyzed. With longer acid exposure, the activity of MMP-7 increased significantly, whereas the activity of MMP-7 decreased significantly with MMP inhibitor treatment (Figure 2D).To further confirm whether the enhanced release of MMP-7 induced after acid exposure play an important role in the cleavage of E-cadherin, the change in MMP-7 and E-cadherin proteins by acid exposure were evaluated in the cultured cells transfected by MMP-7 siRNA. MMP-7 siRNA transfected cells showed a decrease in the levels of MMP-7 protein compared with that of negative control (NC siRNA transfection) and no change in the levels of cellular E-cadherin and sE-cad after acid exposure (Figure 2E, Supplementary Figure S3B,C). These results indicate that MMP-7 may have an important role in the cleavage of E-cadherin following acid exposure.

### 2.3. Confirmation of E-cadherin and MMP-7 Expression in the Human Pharynx

**Figure 3:**
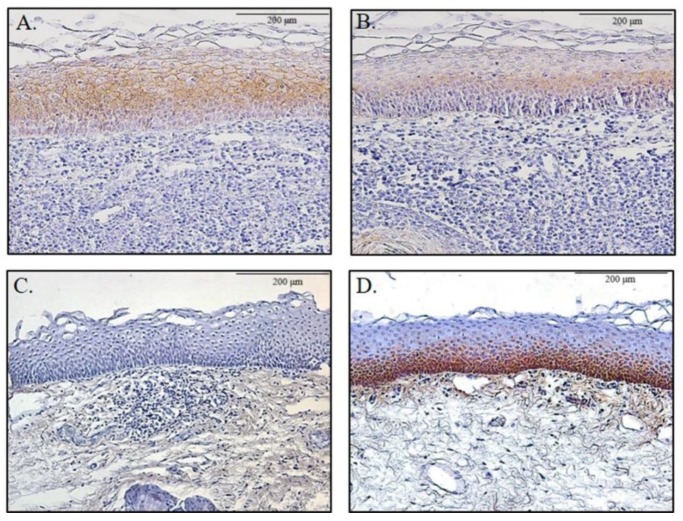
E-cadherin and MMP-7 expression in human pharyngeal mucosa. E-cadherin expression was significantly higher in those with reflux symptom index score of less than or equal to 13 (**A**) compared to those with a reflux symptom index score greater than 13 (**B**). The expression of MMP-7 was significantly higher in those with a reflux symptom index score greater than 13 (**D**) compared to those with a reflux symptom index score of less than or equal to 13 (**C**).

Patients who underwent tonsillectomy were categorized into two groups based on the reflux symptom index score of LPRD. The group with an RSI score higher than 13 showed a significantly lower expression of E-cadherin and higher expression of MMP-7 in the pharyngeal mucosal layer compared to that of the other group with an RSI score ≤ 13 (*p* < 0.05, Table 1) (Figure 3).

## 3. Discussion

The present study demonstrates that changes in E-cadherin in human pharyngeal epithelium by acid exposure may be mediated by MMP-7. Acid exposure for 1 min or 5 min elicited maximal degradation of epithelial E-cadherin without a decrease in the mRNA level of E-cadherin 24 h. Thus, this phenomenon shows that the short-term acid exposures to the mucosa do not destruct epithelial E-cadherin directly through chemical damage but can cause the decomposition of E-cadherin by a mechanism acting for 24 h after the exposure.

When 24 h passed after the epithelial cells were exposed to acid, an increase in sE-cad was examined in the culture media in contrast to the decrease of full-length E-cadherin in the cell portions. Such an increase of sE-cad in the culture media after acid exposure was associated with MMP activity. In particular, duration of acid exposure showed a positive correlation with the increase in mRNA expression and enzymatic activity of MMP-7 along with an increase in transepithelial permeability. As expected, treatment of MMPI alleviated the decrease in E-cadherin levels and the increase in permeability. Furthermore, MMP-7 siRNA-transfected cells elicited no changes in cellular E-cadherin and sE-cad after acid exposure. Consequently, the increase in levels of sE-cad and decrease in levels of cellular E-cadherin were presumed to be the result of MMP activity after acid exposure.

Thus far, 24-h double-probe pH monitoring is well-known as a sensitive and specific diagnostic tool in LPR [1]. Even if there is a debate on the normal limit for the pH monitoring in the literature, the percentage of time at pH less than 4.0 can be used for the diagnosis of LPR [23,24]. Even a single episode of reflux in the proximal laryngeal probe may be considered indicative of LPR [1]. Additionally, the acidic condition at approximately pH 2.0, which can be detected close to the lower esophageal junction is very rare in the pharynx. Previous studies have shown that morphological changes associated with esophagitis were observed due to an acid exposure of pH 2 to the porcine esophagus, but similar changes were observed due to an acid exposure of pH 4 to the porcine larynx. These results indicate that the laryngopharynx may be more sensitive to the acidic and pepsin effects than the esophagus [25]. Actually, patients with a high RSI score can have at least one reflux episode for 24 h in the pH 4.0–4.5 environment [26]. Therefore, in the present study, acid exposure to pharyngeal cells was investigated at pH 4.

E-cadherin is a type-I transmembrane glycoprotein and localizes to the adherens junction and basolateral membrane in various epithelial cells. E-cadherin has an important function in the structural organization of cells in tissues and organs of multicellular organisms [27]. The structure of E-cadherin consists of three domains: (1) a large extracellular domain, (2) a transmembrane segment, and (3) a conserved cytoplasmic domain. The extracellular portion of E-cadherin contains five extracellular cadherin domain repeats that bind to calcium ions to form a stiffened linear molecule [21]. The adhesive function of E-cadherin plays a vital role in epithelial physiology. The fully formed cadherin–catenin complex, with its associated actin filaments, forms the core of the adherens junction, which brings together two apposed plasma membranes with an intercellular gap of only 25 nm [17].

E-cadherin fragments can be generated at several points. MMP cleavage occurs on the extracellular face of the plasma membrane, leading to generation of sE-cad (N-terminal 80-kDa fragment) [28,29,30]. In a previous study with esophageal epithelium from patients with gastroesophageal reflux disease (GERD), the cleavage of E-cadherin was documented in the esophageal mucosa by the presence of the 35-kDa C-terminal fragment of the molecule shown in the Western blots. In addition, serum levels of the 80-kDa N-terminal fragment were found to be significantly higher in GERD patients [18]. Similarly, in the present study that used a primary cell culture of pharyngeal mucosa cells, acid exposure resulted in a decrease in full-length E-cadherin in the cellular component and increased sE-cad in the culture media. Interestingly, when pharyngeal mucosal cells were exposed to pH-4 acid media for less than 5 min and washed out with non-acidic BEBM media, significant decreases in E-cadherin levels without significant changes in E-cadherin mRNA were elicited over a 24-h period after acid exposure. This time-delayed change may indicate an association with another mechanism and may not be due to direct injury from acid exposure. As shown, the cleavage of E-cadherin was related to an increase in MMP-7 expression and enzymatic activity after acid exposure, which may be a reason why the time delayed cleavage of E-cadherin occurred after acid exposure. In addition, the sE-cad may be a negative competitor to membrane-bound E-cadherin that interferes with the homophilic interaction of the full-length E-cadherin homodimers between adjacent cells [31]. Moreover, sE-cad causes the upregulation of major MMPs, and further disruption of E-cadherin and cell-cell junction may be continued [21]. Considering the effect of sE-cad and MMP is maintained for 24 h after washout of acid, single or repeated acid exposure even for short periods can cause symptoms and physical findings consistent with LPRD.

In the present study, immunocytochemical staining showed that intercellular staining of E-cadherin in the cell-cell junctions decreased, and intracellular staining of E-cadherin increased after the 5-min acid exposure in the cells without the MMP inhibitor. Similar to these results, the previous study reported that the cytoplasmic expression of E-cadherin was observed in the laryngeal tissue of patients diagnosed with LPR [32].

A retrograde flow of gastric contents into the laryngopharynx has been proposed as a common etiologic factor of LPRD [2,3]. Therefore, PPIs are empirically prescribed to such patients under the presumption that acid exposure to pharyngeal mucosa is associated with LPRD [1,33,34]. However, a previous systemic review suggests that PPI treatment may offer a nonsignificant clinical benefit over placebo in LPRD [34]. Consequently, LPRD may be associated with other pathophysiologic mechanisms for acid exposure, and a novel targeted drug for LPRD is needed. Our study demonstrated that E-cadherin of pharyngeal epithelium was degraded by the activation of MMP-7 after acid exposure and suggested that E-cadherin could be exploited as a therapeutic target. Therefore, E-cadherin and MMP-7 may be targets for the detection of LPRD and MMPI, and MMP-7 may be useful for additional therapeutic agents for LPRD.

The present study suggested an increase in MMP-7 in acidic environments, but did not confirm the mechanism by which it increases. Previous studies have reported that reactive oxygen species (ROS) increase in acidic environments [35] and regulates extracellular MMP activity through transcription and post-translational mechanisms [36]. In addition, ROS may modulate MMP activity directly through the regulation of MAPK and transcription factors [37]. Therefore, the acid-MMP-7-E-cadherin mechanism may be associated with the specific mechanism of ROS. In future studies, the specific mechanism of ROS on expression and activity of MMP7 should be evaluated.

The present study has some limitations regarding the reliability of data because the evidence of the acid-MMP-E-cadherin mechanism was proven mainly by in vitro study, even if the similar results was examined in the histologic evaluation of pharyngeal mucosa harvested from human volunteers who had tonsillectomy. Therefore, in vivo studies should be performed for a validity of the present study under various conditions. Further investigation is also needed using various other materials such as pepsin and bile, which are suspected to be causes of LPRD.

In summary, acid exposure on pharyngeal cells resulted in a decrease in surface E-cadherin levels and an increase in the levels of the extracellular 80-kDa E-cadherin fragment. The duration of acid exposure was important in the cleavage of E-cadherin, leading to an increase in intercellular permeability. Exposure to acid resulted in the activation of MMP-7, which in turn could elicit further cleavage of E-cadherin. In contrast, the blockage of MMPs could reduce the cleavage of E-cadherin and reduce the loss of mucosal permeability. Given the importance of sE-cad and MMP-7 in the pathophysiologic mechanism of LPRD, blocking MMP-7 may be a novel means of therapy in LPRD patients.

## 4. Materials and Methods

### 4.1. Tissue Preparation

Normal pharyngeal mucosa was harvested from the posterior pillar area of 35 volunteers who underwent tonsillectomy due to tonsillar hypertrophy and sleep problems (13 men and 22 women; age range, 19–51 years). Subjects had no sign of acute inflammation of the pharynx and no history of allergy, smoking, or ongoing drug treatment. Of the 42 samples, primary cultivation was achieved successfully with 35 samples, and the positive ratio was 83.3%. Protocols and experimental design parameters were reviewed and approved by the Institutional Review Board of Korea University Hospital (IRB No. ED15303). Informed consent was obtained from all participants, and all methods were performed in accordance with the relevant guidelines and regulations.

### 4.2. Cell Culture

Human pharyngeal mucosal samples were incubated in 1 mg/mL dispase in Dulbecco’s modified Eagle’s medium/F12 (DMEM/F12) for 1 h at 37 °C in 5% CO_2_. The pharyngeal epithelial cells were then freed by curettage and collected into a 15-mL conical tube. After centrifugation, the cells were rinsed three times with DMEM/F12 and cultured in serum-free bronchial epithelial growth medium (BEBM, Lonza, Walkersville, MD, USA) supplemented with bovine pituitary extract, insulin, hydrocortisone, gentamycin, amphotericin B, retinoic acid, transferrin, triiodothyronine, epinephrine, epidermal growth factor, 100 U/mL of penicillin, and 100 μg/mL of streptomycin [38]. On reaching approximately 70% confluency, the cells were detached with 0.25% trypsin EDTA, washed in DMEM/F12, and resuspended in BEBM kit media in 12-well culture plates (SPL, Seoul, Korea) at approximately 1 × 10^5^ cells/well. Passage 2 of pharyngeal epithelial cells were used in the all experiments. Routine cultures were maintained in a 5% CO_2_ incubator at 37 °C, and the media was changed every 3 days. Staining of the epithelial cell marker cytokeratin was performed to confirm the epithelial cells, and mycoplasma test results showed no abnormalities. Cell morphology was examined using an Olympus CKX41-A32PHP microscope (Olympus, Tokyo, Japan). For statistical data analysis and to ensure reproducibility, each experiment was repeated at least three times using pharyngeal epithelial cells from different patients.

### 4.3. Acid Exposure and MMP Inhibitor Treatment

The confluent pharyngeal epithelial cells were exposed to media adjusted with HCl at pH 4 for 30 s, 1 min, or 5 min. After exposure for each incubation period, the acidic medium was replaced with non-acidic BEBM medium, and the cells were incubated at 37 °C, in 5% CO_2_ for 24 h, washed twice with PBS, and used for experiments.

To investigate the effect of the MMP inhibitor (actinonin, Santa Cruz Biotechnology, Santa Cruz, CA, USA) on acid exposure to pharyngeal epithelial cells, the epithelial cells were pretreated with 10 μM actinonin for 1 h at 37 °C in 5% CO_2_ After treatment with 10 μM actinonin and a pH-4 solution for 30 s, 1 min, or 5 min, the cells were incubated with 10 μM actinonin in non-acidic BEBM media for 24 h. The control cells were treated with non-acidic BEBM media and 10 μM actinonin for the same period. Then, the cells were washed twice with PBS and used for experiments.

### 4.4. Real-Time PCR

Gene expression in the epithelial cells was measured using quantitative real-time PCR. Total RNA was extracted from approximately 5 × 10^5^ cells using Trizol and DNase (Qiagen, Germantown, MD, USA). The prepared cDNA was amplified and quantified using SYBR green master mix (Qiagen) with the following primers: GAPDH, forward 5′-GAG TCA ACG GAT TTG GTC GT-3′ and reverse 5′-TTG ATT TTG GAG GGA TCT CG-3′; MMP-2, forward 5′-TCT CCT GAC ATT GAC CTT GGC-3′ and reverse 5′-CAA GGT GCT GGC TGA GTA GAT C-3′; MMP-3, forward 5′-ATT CCA TGG AGC CAG GCT TTC-3′ and reverse 5′-CAT TTG GGT CAA ACT CCA ACT GTG-3′; MMP-7, forward 5′-TGA GCT ACA GTG GGA ACA GG-3′ and reverse 5′-TCA TCG AAG TGA GCA TCT CC-3′; MMP-9, forward 5′-TTG ACA GCG ACA AGA AGT GG-3′ and reverse 5′-GCC ATT CAC GTC CTT AT-3′; and CDHI for E-cadherin, forward 5′-TGC TCT TGC TGT TTC GG-3′ and reverse 5′-TGC CCC ATT CGT TCA AGT AG-3′. The PCRs were performed using a real-time thermal cycler system (TP800/TP860, Takara, Kusatsu, Shiga, Japan) with 40 cycles of a 2-step reaction consisting of denaturation at 95 °C for 15 s, followed by annealing/extension at 60 °C for 45 s. Data was analyzed using the △*Ct* method.

For investigating the effect of the transcriptional inhibitor, the pharyngeal epithelial cells were pretreated with 5 μg/mL Actinomycin D (Sigma) at 37 °C in 5% CO_2_ for 30 min [39]. After treatment with 5 μg/mL Actinomycin D and a pH-4 solution for 30 s, 1 min, or 5 min, the cells were incubated with 5 μg/mL Actinomycin D in non-acidic BEBM media for 24 h. The control cells were treated with non-acidic BEBM media and 5 μg/mL Actinomycin D for the same period. Then, the cells were washed twice with PBS and used for real-time PCR of MMP-7.

### 4.5. Western Blot

For the western blot analysis, acid-treated, acid/MMP inhibitor-treated or acid/MMP-7 siRNA-transfected human pharyngeal epithelial cells were collected by scraping the cells to maintain the cell-cell interaction without single-cell dissociation, and the supernatant of each well was concentrated to equal volume using Centricon (3 kDa cutoff, Merck Millipore, Billerica, MA, USA) at 3000× *g* for 40 min at 4 °C. Then, each sample was mixed with 5× Laemmli buffer and 5% β-mercaptoethanol and boiled for 10 min. The extracts were separated on 8% SDS-polyacrylamide gels and transferred to nitrocellulose membranes for 30 min at 350 mA in transfer buffer (25 mM Tris, 192 mM glycine, 0.1% SDS and 20% methanol, pH 8.3). Nonspecific binding sites on the membranes were blocked in 5% skim milk for 90 min at room temperature. The membranes were then incubated overnight at 4 °C with a 1:1000 dilution of E-cadherin (clone number H-108, catalog number sc-7870, Santa Cruz) or MMP-7 (catalog number AV46075, Sigma-Aldrich, St. Louis, MO, USA) or a 1:2000 dilution of β-actin antibody (clone number C4, catalog number sc-47778, Santa Cruz) for loading control in blocking solution. Next, the membranes were incubated with a 1:1000 dilution of the appropriate anti-rabbit (catalog number sc-2004, Santa Cruz) or anti-mouse antibody (catalog number sc-2005, Santa Cruz) in blocking solution. Blots were visualized using the chemiluminescence kit (Santa Cruz), which was captured with ChemiDoc (Bio-Rad Laboratories, Hercules, CA, USA).

To evaluate the expression of E-cadherin, it is important to preserve the cell-cell junctional adhesions. Therefore, instead of using protein quantitative analysis after single cell dissociation, the difference of E-cadherin expression was semiquantitatively estimated based on the β-actin expression in each sample. And the supernatant amount of protein in each sample was determined according to a bicinchonic acid (BCA) protein assay, using bovine serum albumin as a standard, and equal amounts of protein were loaded on the gel.

### 4.6. MMP-7 Enzyme Substrate Assay

For the activity measurement of MMP-7, the culture media from acid-treated or acid/MMP inhibitor-treated pharyngeal epithelial cells were concentrated using Centricon spin tubes (3 kDa cutoff, Merck Millipore) at 3000× *g* for 40 min at 4 °C. The concentrate was incubated with 10 μM APMA (4-aminophenylmercuric acetate) in assay buffer for 1 h at 37 °C. After the addition of dilute MMP-7 substrate (5-FAM/QXL^TM^ 520 FRET peptide) (SensoLyte 520 MMP-7 assay kit, Anaspec Inc., Fremont, CA, USA) 1:100 in assay buffer and incubation for 1 h at 37 °C, the samples were read at an excitation wavelength of 490 nm and emission of 520 nm (SOFTMAX PRO v5 software, Molecular Devices, Sunnyvale, CA, USA) with a SpectraMax M2^e^ plate reader (Molecular Devices).

### 4.7. Silencing of MMP-7 by Small Interfering RNA (siRNA)

To analyze the effect of MMP-7 inhibition, siRNA directed against either human MMP-7 or negative control (NC, scrambled) were purchased from OriGene Technologies Inc. (Rockville, MD, USA). The cultured human pharyngeal epithelial cells were grown in 12-well plates and transfected with MMP-7 siRNA (sequence: rCrCrArUrArGrGrUrCrCrArArGrArArCrArArUrUrGrUrCTC) or NC (at a final concentration, 10 nM) using Lipofectamine (Invitrogen, Carlsbad, CA, USA), according to the manufacturer’s instructions. At 36 h after transfection, the cells were treated with acid for 30 s, 1 min, or 5 min, incubated for 24 h, and the harvested cells or culture media were used for western blot analyses.

### 4.8. Immunocytochemistry Analysis

Cells were cultured on cytoslides (Marienfeld-Superior, Lauda-Königshofen, Germany) and treated with acid or acid/MMP inhibitor as mentioned above. Immunocytochemical analysis was performed after culturing in non-acidic BEBM medium or in non-acidic BEBM medium supplemented with MMP inhibitor for 24 h after acid exposure. Cells were fixed with 4% glutaraldehyde for 30 min and blocked for 1 h at room temperature with goat serum (Vector Laboratories, Burlingame, CA, USA). The cells were then incubated with rabbit polyclonal antibody against E-cadherin (1:50, Santa Cruz) or cytokeratin (1:100, clone number H-240, catalog number sc-15367, Santa Cruz) overnight at 4 °C. During the next day, the cells were treated with biotinylated anti-rabbit IgG (H+L) secondary antibody, which was made in goat (Vector Laboratories) in PBS (1:400) for 60 min at room temperature. After washing the cytoslides with PBS, antigen-antibody complexes were detected using an avidin-biotin complex detection system (Vectastain ABC Kit, Vector Laboratories). The cytoslides were stained using the DAB Substrate kit (Vector Laboratories), rinsed in water, briefly counterstained with Mayer’s hematoxylin and washed again in water. After mounting on glass slides, the cytoslides were examined using an Olympus BX51 microscope. Pictures were captured and controlled using an Olympus DP72 and DP2-BSW. Immunostaining of E-cadherin was evaluated in five microscopic fields (×200) of three different samples. To quantify the E-cadherin expression, the number of E-cadherin-stained cells throughout the cell membrane was counted. Then, the semiquantitative score was calculated as the percent of the number of the stained cells per total number of cells in each microscopic field.

### 4.9. Transepithelial Permeability Analysis

Human pharyngeal epithelial cells were seeded at a density of 1 × 10^5^ cells/cm^2^ on 12-transwell culture plates with 0.4-μm polyester filters (SPL). The transepithelial permeability test was performed 24 h after acid exposure with/without MMP inhibitor treatment as mentioned above. Fifty microliters of 100 μM rhodamine B isothiocyanate (RITC)-labeled Dextran 70S (Sigma) was added to the top chamber of the transwell plates. For the next 3 h, 50 μL of the media samples were collected from the bottom compartments at 30-min intervals and analyzed on a SpectraMax M2^e^ plate reader (Molecular Devices) with an excitation wavelength of 530 nm and emission of 590 nm (SOFTMAX PRO v5 software, Molecular Devices) [40].

### 4.10. Histologic Evaluation in Human Pharyngeal Tissue According to Reflux Symptom Index

Pharyngeal mucosa was harvested from a total of 22 volunteers who had tonsillectomy for chronic tonsillitis. The reflux symptom index (RSI) was surveyed using the following indicators: (1) hoarseness or a problem with the voice; (2) clearing of the throat; (3) excess throat mucous or postnasal drip; (4) difficulty swallowing food, liquids, or pills; (5) coughing after eating or after lying down; (6) breathing difficulties or choking episodes; (7) troublesome or annoying cough; (8) sensations or something sticking in the throat; and (9) heart burn, chest pain, indigestion, or stomach acid. The scale for each individual item ranges from 0 (no problem) to 5 (severe problem) and then the final RSI score was calculated by total sum of each score, with a maximum total score of 45. According to the diagnosis criteria for LPRD, patients were categorized into two groups; (1) patients with an RSI score ≤ 13 (*n* = 8), and (2) patients with an RSI score higher than 13 (*n* = 14) [4].

The paraffin sections (4 μm) of human pharyngeal tissues were cut, deparaffinized, and treated with 0.03% H_2_O_2_ in methanol for 10 min to quench endogenous peroxidase activity. After washing in PBS, the sections were then incubated overnight at 4 °C with E-cadherin antibody (1:200, Santa Cruz) or MMP-7 antibody (1:200, Sigma). During the next day, the sections were treated with biotinylated anti-rabbit IgG (H+L) secondary antibody (Vector Laboratories) in PBS (1:400) for 2 h at room temperature. Then, antigen-antibody complexes were detected using an avidin-biotin complex detection system (Vectastain ABC Kit, Vector Laboratories) and a DAB Substrate Kit (Vector Laboratories). After counterstaining with Mayer’s hematoxylin, the sections were examined using an Olympus BX51 microscope. The pictures were captured in Olympus DP72 and DP2-BSW and staining intensity was compared semiquantitatively using the Image J program. Then, the patients were classified as a weak intensity group (no or mild stain intensity) and strong intensity group (moderate or strong stain intensity). The Institutional Review Board (IRB No. ED15303) of Korea University Anam Hospital approved all of the protocols and study design, and all patients gave written informed consent.

## Figures and Tables

**Table 1:** Expressions of E-cadherin and MMP-7 according to reflux symptom index.

Markers	13 or Lower than 13 (*n* = 8)	Higher than 13 (*n* = 14)	*p*-Value
E-cadherin			0.007
Weak	0	8	
Strong	8	6	
MMP-7			0.004
Weak	6	2	
Strong	2	12	

Staining intensity scores were as follows: weak = no staining and mild stain intensity, strong = moderate and strong stain intensity. Statistical analysis was performed using SPSS version 20.0 (IBM SPSS, Armonk, NY, USA). Statistical significance was considered to be *p* < 0.05.

## Supplementary Materials

Supplementary materials can be found at https://www.mdpi.com/1422-0067/20/21/5276/s1.
