# Supplementary material for: Role of Matrix Metalloproteinases 7 in the Pathogenesis of Laryngopharyngeal Reflux: Decreased E-cadherin in Acid exposed Primary Human Pharyngeal Epithelial Cells
Source: Int J Mol Sci. 2019 Oct 24;20(21):5276. doi: 10.3390/ijms20215276 (PMC6862869; doi:10.3390/ijms20215276)
Supplement: Supplementary file 1 [file ijms-20-05276-s001.pdf]

Supplementary Materials

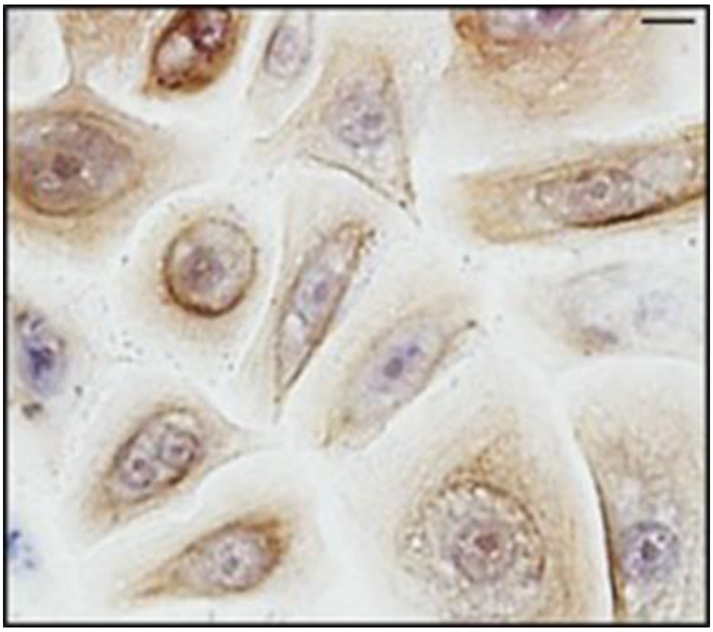

Supplementary Figure S1. Cytokeratin immunocytochemical stain of primary pharyngeal epithelial cells. Most of the pharyngeal epithelial cells show cytokeratin expression (bar, 50 um).

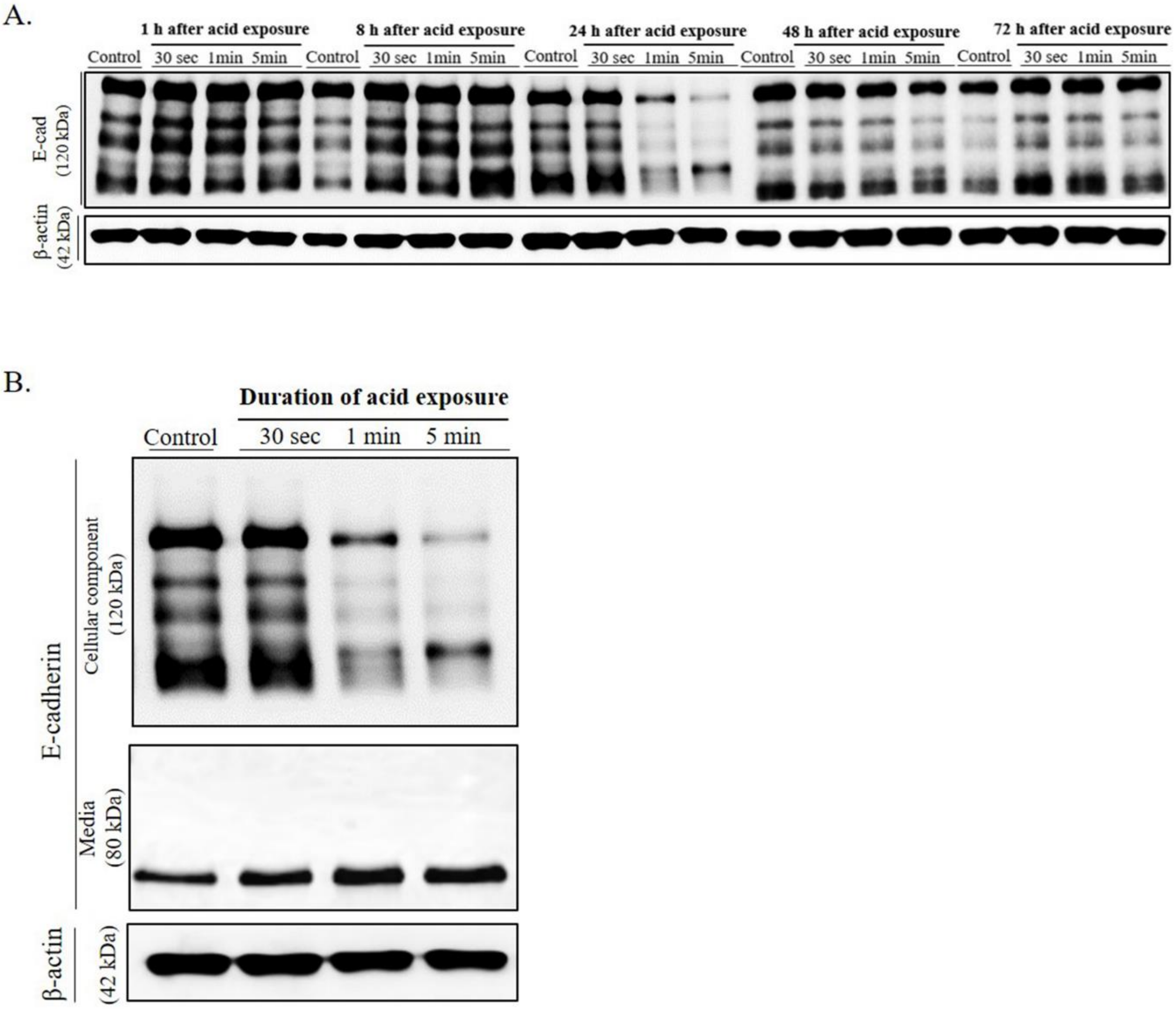

Supplementary Figure S2. E-cadherin cleavage in human pharyngeal mucosal epithelial cells exposed to acidic media (Full-length blots/gels of Figure 1).

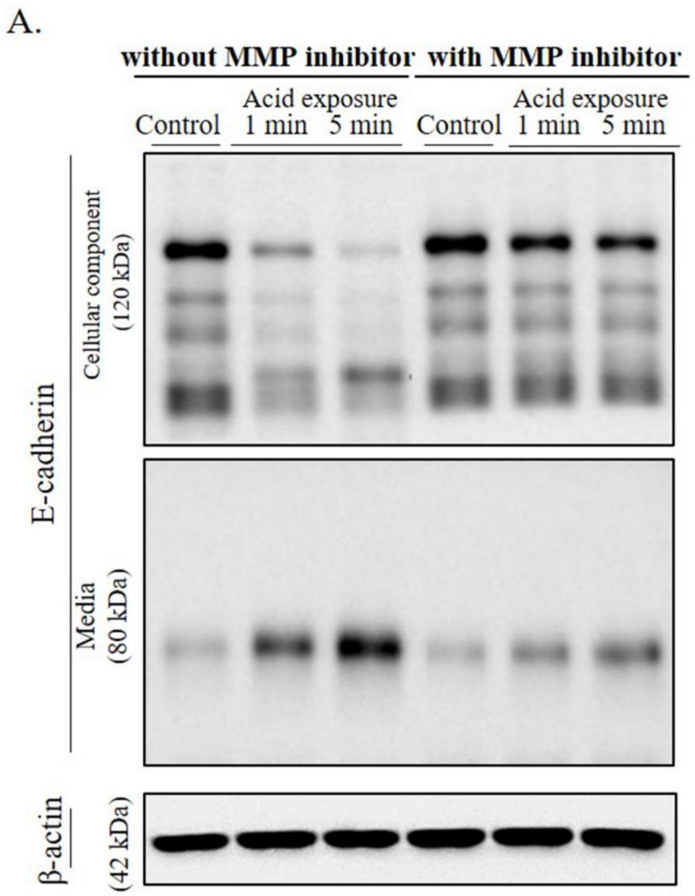

Supplementary Figure S3. Effect of MMP inhibitor on changes in E-cadherin (Full-length blots/gels of Figure 2).

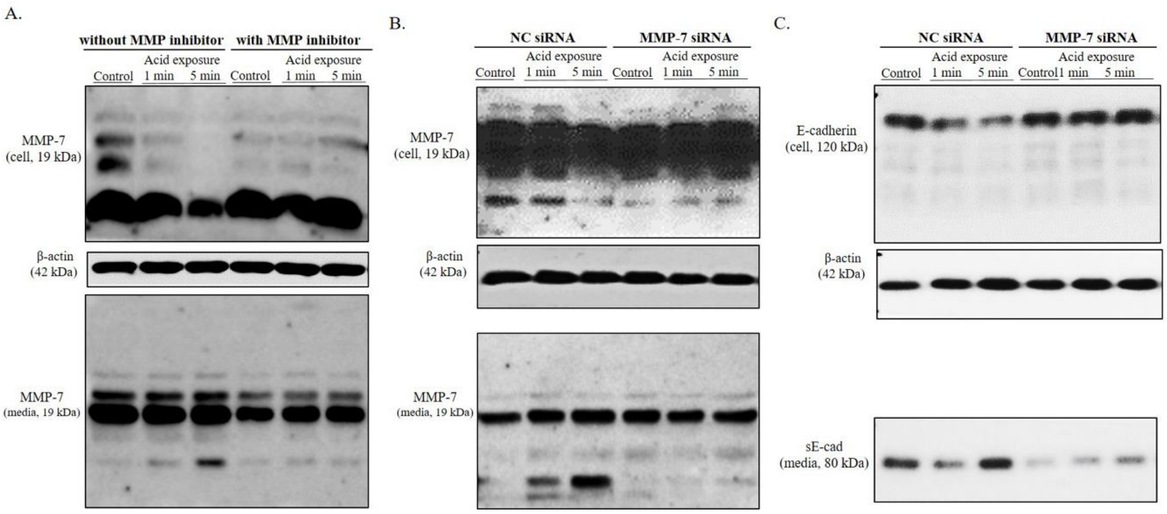

Supplementary Figure S4. Effect of MMP inhibitor and MMP-7 knockdown on changes in MMP-7 (Full-length blots/gels of Figure 4).
